# Scale-Up of Alfalfa (*Medicago sativa*) Protein Recovery Using Screw Presses

**DOI:** 10.3390/foods11203229

**Published:** 2022-10-15

**Authors:** Mikkel Hansen, Christina Albers Andersen, Peter Ruhdal Jensen, Timothy John Hobley

**Affiliations:** National Food Institute, Technical University of Denmark, DK-2800 Kgs. Lyngby, Denmark

**Keywords:** green proteins, sustainability, alternative proteins, green food

## Abstract

As a consequence of the increased demand for proteins for both feed and food, alternative protein sources from green plants such as alfalfa (*Medicago sativa*) have come into focus, together with methods to recover these proteins. In this study, we have investigated the use of screw presses for protein recovery from alfalfa at laboratory and pilot scale. We found that using a pilot scale screw press, with a working pressure of 6 bar, 16% of the total protein was recovered in one pressing, and that after rehydrating and repressing the alfalfa up to ten times, 48% of the total protein could be recovered. The green alfalfa protein concentrate was analyzed for total protein, amino acid profile, protein digestibility, color, ash, fiber and fat content. It was found that repetitive pressings lowered the digestibility of the protein pool and reduced the total protein concentration due to dilution. To achieve the best quality protein at the highest concentrations, it is recommended to press the alfalfa no more than twice, which results in an alfalfa protein concentrate with more than 32% soluble protein and greater than 82% digestibility.

## 1. Introduction

It is currently estimated that the production of protein needs to increase by 70% before 2050 to meet the demand for a growing and more wealthy population in the world [1]. As a consequence, new protein sources for animal and human diets must be developed, with low carbon footprints and high protein yields from the available arable land [1]. 

One of the potential new protein sources could be alfalfa, a perennial plant, grown world-wide as a feed for ruminants, pigs and hens. It is favorable as a protein crop with a protein yield per acre of land, which is up to 10 times higher than soybean and it has a high tolerance to lack of water due to its deep roots [2,3,4]. The deep root network also prevents washing out of nutrients from the soil to the aquatic bio-systems surrounding many fields [4]. Alfalfa also contains all essential amino acids. Furthermore, due to its ability to fix nitrogen from the air to the soil, it has been used for decades as a natural fertilizer. Recently alfalfa has also been suggested as a potential protein source in the human diet.

The leaves and sprouts of alfalfa have already been used in salads and soups and the plant as a whole is approved as safe to consume in the novel food catalogue [5,6]. It is therefore of interest to isolate an alfalfa protein concentrate, which could potentially be more sustainable than other plant protein alternatives such as soy. For animal feed, alfalfa is typically harvested 4–5 times per year and it is used to produce silage or it is transported directly to a biorefinery for protein extraction. The proteins are commonly extracted from either the whole plant or parts of it. A unit operation for mechanical separation of the plant material is used, followed by a press to produce a protein rich green juice. The green juice is then processed in various ways to isolate the proteins. They are then dewatered by centrifugation, followed by a drying step, yielding an alfalfa protein concentrate (APC) for animal feed supplementation [4,6,7,8,9,10]. The main protein found in alfalfa is RuBisCO which is highly soluble and constitutes up to 70% of the soluble proteins found in alfalfa [11]. RuBisCO consists of a large subunit (55 kDa) and eight small subunits (15 kDa) [12].

With respect to human consumption, the green APC has been approved as a novel food supplement since 2009, with a daily intake of up to 10 g. Besides its favorable amino acid composition, it also contains several unsaturated fatty acids and vitamins, B, C, D, E and K [6]. Attempts to maximize yield can often negatively affect digestibility and functionality due to the processing conditions used. In 1972, Knuckles et al. investigated repetitive pressing for the production of alfalfa protein concentrate. Their study only investigated pressing up to three times and with a focus on using the protein obtained as animal feed [7]. Given the limitations and the age of the work of Knuckles et al., it is important to move the state of the art forward if protein from green biomass is to meet the demand for increased plant-based protein for food applications. In order for the technology to mature, it is important to understand how processing affects the quality of the protein produced and how to scale-up. The aim of the current study, therefore, was to examine how to maximize the yield of green protein from alfalfa using two different screw presses. Repetitive sequential pressing up to 10 times was conducted and it was determined if this had a negative impact on the properties of the protein concentrate. 

## 2. Materials and Methods

### 2.1. Production of Alfalfa Protein Concentrate

**Figure 1:**
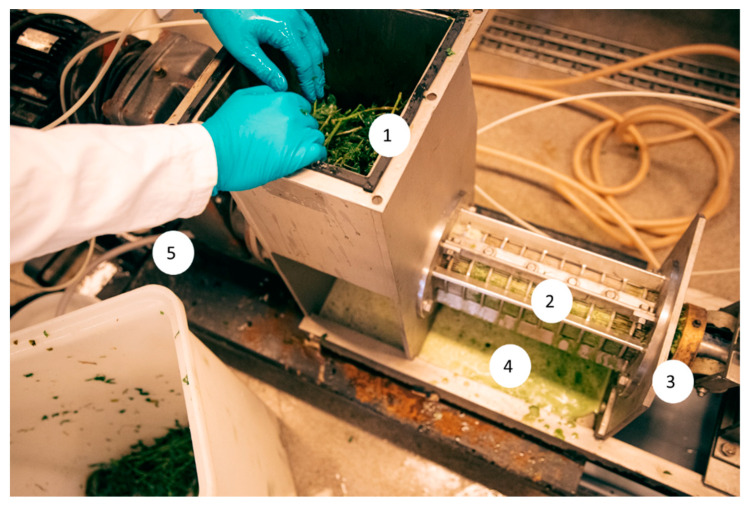
Pressing of alfalfa on Vincent CP-4 single-screw press. 1: Feeding with fresh alfalfa, 2: Screw and 0.5 mm filter matrix, 3: Discharge of pulp, 4: Discharge of the green juice, 5: Collection of the green juice. Photo: Flemming Leitorp.

Alfalfa was harvested in spring 2019, on the island of Fyn in Denmark, manually by scythe. It was transported directly to the laboratory where it was stored at −20 °C, until it was further processed. Before pressing, the alfalfa was thawed by submerging it in cold tap water. It was then drained until it had reached the weight it had before thawing and had equilibrated to room temperature (20–22 °C). The thawed alfalfa was processed at room temperature, either through a Vincent CP-4 single-screw press (Vincent Corporation, Tampa, FL, USA) [13] (Figure 1) or an Angel Juicer S8500 twin-screw press (Angel Juicer, Seoul, South Korea) used with its standard filter. For the Vincent CP-4, the length of the cylinder and screw were 800 and 540 mm, respectively, with a diameter of 50 mm and the pitch of the screw was 50 mm. It was operated at 10 rpm with a 0.5 mm filter. The press has a maximum capacity of 68–227 kg/h. The pressure on the discharge cone was 6 bar, which was the maximum possible. During setup of the machine, lower pressures were seen to give a lower yield of green juice, but they were not systematically investigated. 

**Figure A1:**
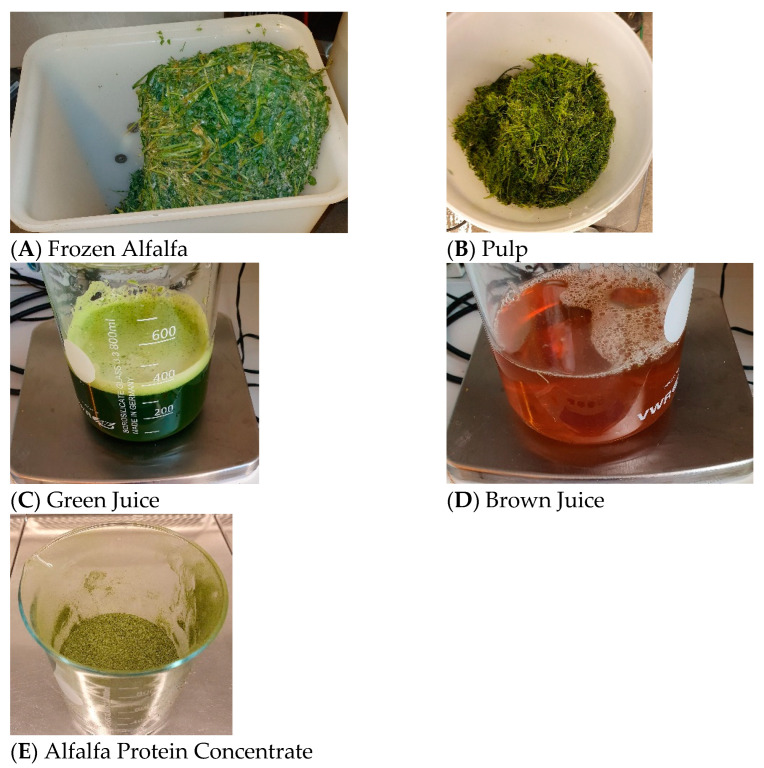
Examples of different fractions in the production of alfalfa protein concentrate.

Pressing of the alfalfa resulted in two fractions: A pulp and a green juice. The green juice was collected immediately and had the pH lowered to 3.5 with 3 M HCl. It was then centrifuged at 2500× *g*, for 15 min at 4 °C with a Thermo Scientific Multifuge X3R (Waltham, MA, USA), within 20 min from the pH adjustment. After centrifugation, two fractions were obtained: A green pellet and a supernatant, the weights of which are presented in Table A1, Appendix A. The green pellet was then freeze-dried using a Thermo Scientific Heto Drywinner DW8 (Waltham, MA, USA). The process started at −20 °C with a 1 °C/h increase until the temperature reached 20 °C. The temperature was then held constant, until no decrease in weight was observed. This resulted in a green protein rich powder called alfalfa protein concentrate (APC). Examples of the fractions obtained can be seen in Figure A1, Appendix A. After the first press, the alfalfa pulp was re-hydrated with an amount of tap water corresponding to two times the weight of the pulp produced, and it was then processed again as described above. This was repeated 10 times. The details of the amounts used are given in Table A1, Appendix A. Due to the mechanical forces in both of the screw presses, it can be expected that the temperature within would increase, however there was no provision for cooling in the presses used here. The temperature of the green juice was not observed to increase significantly above room temperature during the period of pressing and was therefore not accurately measured. After freeze-drying, a sample of APC from each press was checked for dry weight content, as described in the section below. The moisture content of the freeze-dried APC was found to be zero. 

### 2.2. Dry Matter and Ash Content

Dry matter content was determined by first placing dry pre-weighed crucibles containing approximately 5 g of APC each in an oven at 104 °C overnight. The crucibles were then placed in a desiccator to cool before weighing. The dry matter content was then calculated from the loss in weight. The crucibles were then placed in a muffle furnace at 600 °C overnight and the ash content was determined from the loss in weight. With respect to the raw alfalfa, a known weight of approximately 200 g of frozen plant material was used to get a representative sample. It was dried to constant weight at 104 °C in an oven and the moisture content was determined by difference. The dried alfalfa was then homogenized by blending in a kitchen machine (KVL 6300, Kenwood, Yokohama, Japan). From the dried homogenized powder, three samples of around 3 g each were transferred to pre-dried crucibles. They were dried until constant weight at 104 °C, before being treated as described above, to determine the ash content.

### 2.3. Protein, Insoluble and Soluble Fiber and Available Carbohydrates

Protein content was determined using the DUMAS combustion method (rapid MAX N exceed, Elementar, Germany), using a nitrogen-protein conversion factor of 6.25 [14].

Fiber and carbohydrate composition were measured with the “Available carbohydrate/dietary fiber assay kit” (Megazyme, Ireland) [15]. In brief, the procedure consisted of 4 steps:

- Available carbohydrate determination through α-amylase, protease and amyloglucosidase incubation.
- Filtration with 96% ethanol to determinate the soluble fiber fraction.
- Protein and ash determination.
- Calculation of the insoluble fiber fraction from the equation below:

(1) $$Insoluble\;fiber\left(\%\right)=\frac{Weight_{after\;step\;2}-Protein-Ash-Blank}{Weight_{original}}\cdot 100$$

Filtration was performed with 47 mm diameter microglass fiber filters (Thermo Fisher Scientific, Waltham, MA, USA). To ensure that the filter paper did not increase the fiber content of the sample, an extra blank sample was run in duplicate, which showed that the filters did not affect the results. 

### 2.4. Preparation of Protein for SDS-PAGE and Bradford Soluble Protein Analyses

Solubilisation of the protein was done by taking 0.1 g of APC and mixing it with 0.9 mL of 0.1 M phosphate buffer (pH 8.00). It was vortexed for 20 s before being placed in a laboratory shaker where it was mixed for 10 min at 1000 rpm (TS-100C, Biosan, Riga, Latvia). After shaking, the samples were centrifuged for 5 min, at 10,000× *g* and room temperature (Microcentrifuge, Ole Dich, Hvidovre, Denmark) and the supernatant was used for SDS-PAGE or the Bradford assay.

### 2.5. SDS-PAGE

All reagents and equipment used in these analyses were from Biorad (Hercules, CA USA). 10 µL of sample prepared as described above, was mixed together with 5 µL of 4× Laemmli buffer, 4.75 µL of milliQ water and 0.25 µL β-mercaptoethanol. The solution was then incubated for 10 min at 95 °C in a TS-100C heating block (Biosan, Riga, Latvia). 10 µL of the incubated sample was then loaded into a well in a 4–20% Mini-PROTEAN^®^ TGX gel, where the first well was loaded with 5 µL of Precision Plus Standard ladder, before running the gel (140 V, 400 mA, 50 min). The gels were then washed in milliQ water and stained for 1 h (Coomasie R-250). The gels were destained by first rinsing them in milliQ water and then they were left in milliQ water for 3 h; this was repeated three times. The gel was then scanned using a ChemiDoc XRS+ System.

### 2.6. Soluble Protein by Bradford

Soluble protein was measured using the Pierce Coomasie Plus Bradford kit following the instructions from the manufacturer for using microwell plates [16]. All analysis was conducted in triplicate on an Infinite M200 Pro plate reader (TECAN, Männedorf, Switzerland).

### 2.7. Protein Digestibility

Digestibility of the APC was determined following the procedures described by Saunders et al. 1973 [17]. In brief, 1 g of APC was suspended in a 50 mL centrifuge tube with 20 mL of 0.1 N HCl and it was mixed with 1 mL of 0.01 N HCl containing 50 mg of pepsin. The solution was incubated at 37 °C for 48 h with gentle shaking, followed by centrifugation at 2500× *g*, for 15 min, at 4 °C using a Thermo Scientific Multifuge X3R (Waltham, MA, USA). The supernatant was discarded, and the solids were resuspended in 10 mL of deionized water and 10 mL of phosphate buffer (pH 8) containing 5 mg of trypsin. The solution was incubated at 23 °C, with gentle shaking for 16 h, followed by centrifugation (2500× *g*, for 15 min at 4 °C). The supernatant was discarded, and the solids were washed with 30 mL of deionized water followed by centrifugation (2500× *g* for 15 min at 4 °C) and the supernatant was removed. The solids were filtered through a 1.2 µm pore size nitrogen free filter, air dried and analyzed for total nitrogen together with the filter paper. The digestibility was determined by comparing the total protein content before digestion with the total protein in the digested solid material using the DUMAS combustion method.

### 2.8. Amino Acid Analysis

Amino acid (AA) analysis was performed using the EZ:faast amino acid kit (Phenomenex, Torrance, CA, USA). Analysis was done on APC from press 1, 5 and 10 in duplicate. Acid hydrolysis of the APC was done first, by boiling 30 mg of sample in 0.5 mL of 12M HCl for 18 h in an oven at 104 °C. After hydrolysis, the samples were filtered through a 0.22 µm pore size sterile filter and processed according to the instructions for the assay [18]. The hydrolysate was analyzed by liquid chromatography using mass spectrometry (LC/MSD Trap, Agilent, Santa Clara, CA, USA) with an EZ:faast 4u AAA-MS Column (250 × 3.0 mm) (Phenomenex, Torrance, CA, USA). 

### 2.9. Total Fat Analyses

Total fat was determined by using the Rapid NMR Fat Analyzer (CEM, Matthews, NC, USA) with the Powder method. All analyses were performed in duplicate.

### 2.10. L-a-b Color Measurement

L-a-b color was determined by placing around 1 g of APC from each respective press under a small glass plate. The sample was measured in triplicate through the glass plate using a LC 100 spectrocoloriometer (Lovibond, Amesbury, UK).

### 2.11. Statistical Analysis

Each analytical result is reported as the mean value of three replicate sample measurements, except where stated. Standard deviations and statistical differences were analyzed using MS Excel. Differences between the means of samples were analyzed by a single factor ANOVA test with least significant difference (LSD) test at a probability of 0.05.

## 3. Results and Discussion

The overall aim of this work was to examine how to maximize the yield of green protein from alfalfa using a screw press and to determine if this had a negative impact on the properties of the protein concentrate. As part of this, we examined whether scale-up from a laboratory based twin-screw press to a pilot scale single-screw press would affect the yield.

### 3.1. Comparison of Single- and Twin-Screw Presses for Extraction of APC

The small twin-screw press and large single-screw press were used to process 4.9 kg and 50.8 kg, respectively, of alfalfa in the first press, which amounted to a total input of 211 g and 2189 g of protein (derived from Table 1), respectively. The resulting pulp was then re-pressed 10 times. In Table 1, approximately 14.3% of the wet weight is unaccounted for, which is speculated to be soluble and insoluble fiber. 

**Figure 2:**
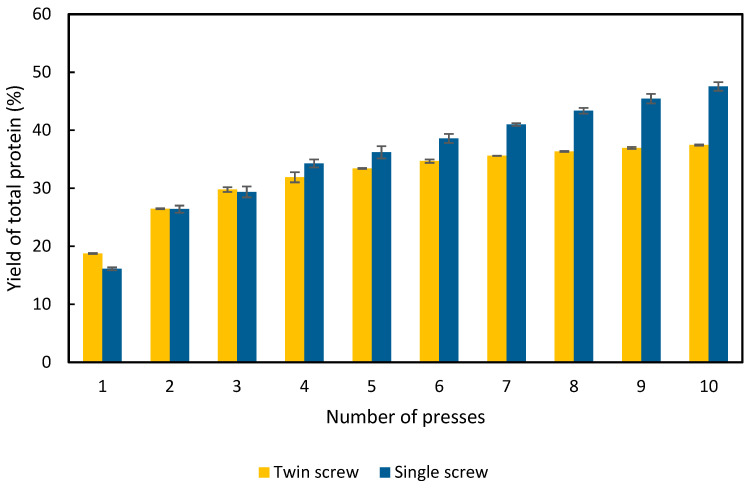
Cumulative yield of protein in the APC as a percentage of the total protein content in the raw alfalfa used in the experiment. Results are from the twin-screw and single-screw press; error bars show standard deviation, n = 3.

The results, presented in Figure 2, show that the protein content of the resulting APC was highest from the twin-screw press in the first two presses, and higher than the single-screw press. In contrast, the single-screw press produced the highest protein concentration in the third press. This difference in press behavior is thought to be due to the design of the respective extractors. The smaller twin-screw press has a section of its screw macerating the product before the extraction occurs, whereas the single screw does not. As a result, it is speculated that the alfalfa plant was not macerated sufficiently before the third press in the single-screw process. After 10 presses, 37% of the total protein was extracted by the twin-screw press (Figure 2). In contrast, 48% was extracted by the single-screw press (Figure 2). Both screw presses extracted approximately 25% of the total protein after the second press. The yield of the twin-screw press stagnated after 6 presses, whereas the single screw continued to extract APC up to the tenth press. Nevertheless, more than half of the protein is not released by either of the presses. 

RuBisCo constitutes up to 70% of the soluble proteins in the alfalfa plant [10], and soluble proteins constitute ca. 33% of the total protein content in alfalfa (including stalks and leaves) [19]. In this work, we have used the entire harvested plant (i.e., including stalks and leaves). Therefore, it is not unexpected that the yield of protein is 50%. The composition and distribution of proteins in the plant is dependent on many factors, such as the harvest point and the alfalfa variety. Nevertheless, it is known that the stems have roughly only half as much protein, compared to the leaves [20]. Therefore, when compared with experiments using only proteins obtained from the leaves, a lower yield is to be expected. Given that 33% of the proteins in alfalfa are soluble, and that this value was reached during the fourth press (Figure 2), it is to be expected that some insoluble proteins are also being extracted by the screw press, which might affect the digestibility and quality of the protein concentrate.

**Figure 3:**
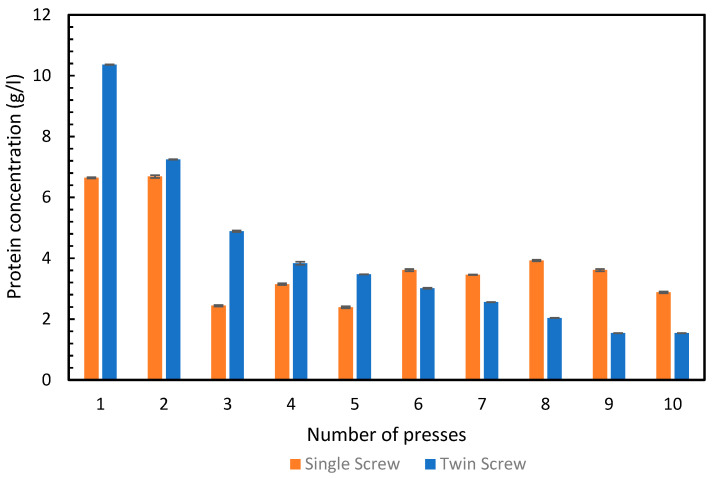
Concentration of total protein (g/L) in the green juice obtained from press 1–10 from the single- and twin-screw presses. Error bars show standard deviation, n = 3.

We obtained 6.6 g/L of total protein from the green juice in the first press and 6.7 g/L in the second press, with the single-screw. All the remaining presses from the single-screw, had significantly lower protein concentrations in their respective green juices, illustrated in Figure 3. In contrast, the twin-screw press produced a green juice with 10.4 g/L protein in the first press and 7.2 g/L in the second press. The concentration continued to drop in all of the further rounds of pressing. This corresponds well with the pattern observed in Figure 3, where it was observed that after the second press, less protein was extracted with the twin-screw press compared to the single-screw press. 

### 3.2. Soluble Protein and Protein Profile by SDS-PAGE

**Figure 4:**
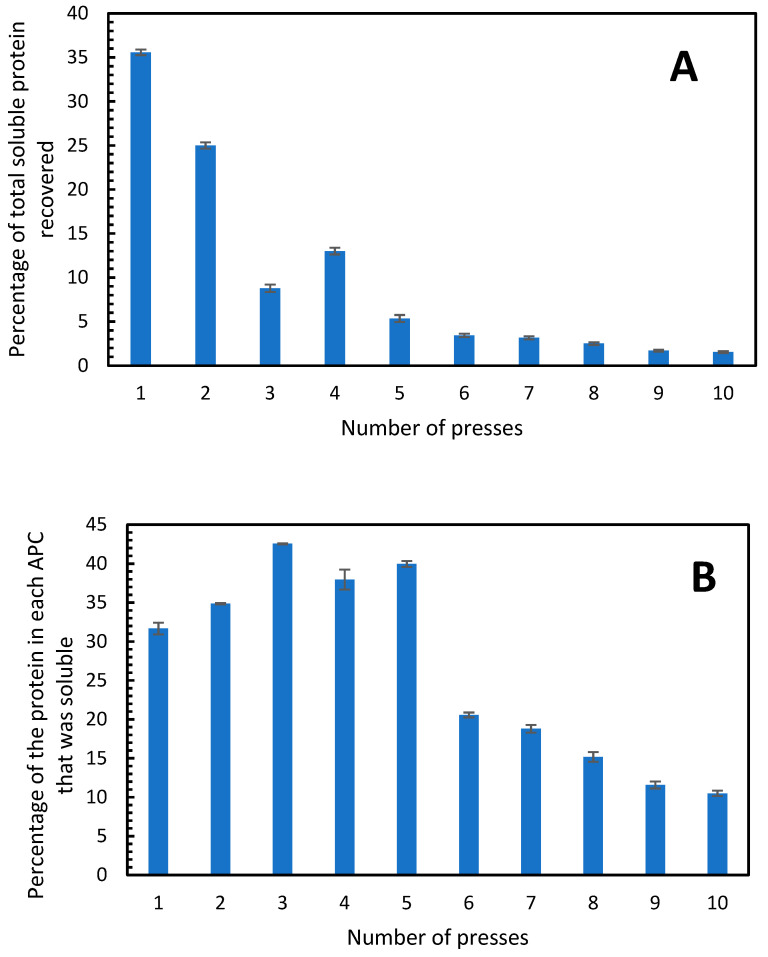
(**A**) Percentage of the total soluble protein in the fresh alfalfa that was present in the APC produced from each press. (**B**) Percentage of soluble protein in each APC fraction. The single-screw press was used. Error bars show standard deviation, n = 3.

The amount of soluble protein in the APC from each press was determined and it was found that 35% of the total possible soluble protein was extracted in the first press, 25% in the second press and in the third and fourth press only 8 and 13% of the total soluble protein was extracted, respectively (Figure 4A). As speculated above, the 50% yield of protein extracted from the alfalfa was likely due to recovery of soluble and insoluble protein, given that only ca. 33% of the total protein in alfalfa is soluble. Therefore, the proportions of soluble and insoluble proteins in APC from the single-screw press were determined. In the APC from the first press, the proportion of soluble protein was close to 33%, which was further enriched to 42.5% in the APC from the third press (Figure 4B). This is consistent with the results in Table 2 where the third press resulted in the highest percentage of protein in the APC. The APC produced from each of the first five presses had a proportion of soluble protein, which was almost twice as high as that in the second five presses (Figure 4B). This indicates that when the alfalfa is pressed more than 5 times, the APC produced contains greater and greater proportions of insoluble protein. A total of 1041 g of protein was extracted in the 10 presses (from a total possible protein amount of 2189 g), from this, 314 g was soluble, which corresponds to 30.1%. Therefore, in order to generate an APC with the highest proportion of soluble protein, a maximum of five pressings should be used. 

**Figure 5:**
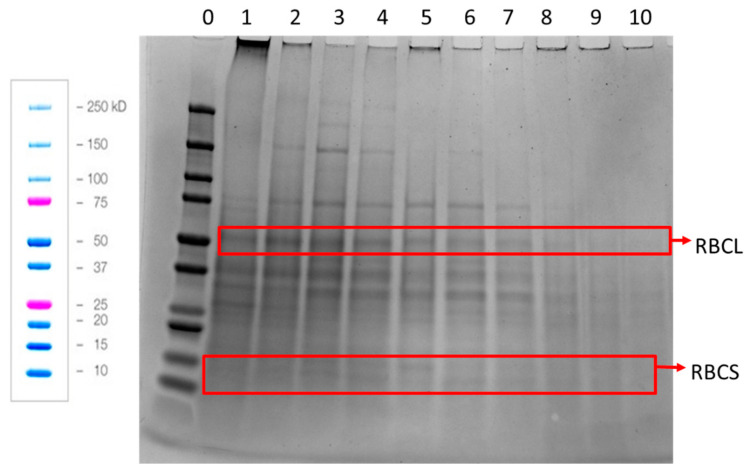
Reducing SDS-PAGE of the APC obtained from press 1–10 from the single-screw press. Lane 0, molecular weight standards. Lanes 1–10, APC from presses 1–10, respectively. RBCL = Large RuBisCO subunit, RBCS = Small RuBisCO subunit. Blue and pink colored bands are from Biorad’s instructions showing how the standards are expected to run. The same mass of APC from each pressing was used to prepare the sample loaded onto each well of the gel.

When the protein composition of the APC was examined by reducing SDS-PAGE, it could be seen that the APC contained more than 20 proteins (Figure 5). The most distinct bands were at ca. 150, 75, 55, 37, 30 and 14 kDa. The small and large RuBisCO subunits are identified at 14 and 55 kDa, respectively [12]. The other bands are speculated to be enzymes which hydrolyze active and non-structural proteins, as suggested by Yakhlef et al., 2020 [21]. Interestingly, it could be observed that the pattern of the protein bands changed from press to press. The large RuBisCO subunit is visible in press 1–8 and the smaller subunit is only visible in press 2–7. Furthermore, the second, third and fourth pressings had three bands with molecular weights of 150 kDa and higher, which were not observed in the other pressings. These bands are most clearly visible for pressing 3. 

### 3.3. Amino Acid Profile of APC

**Figure 6:**
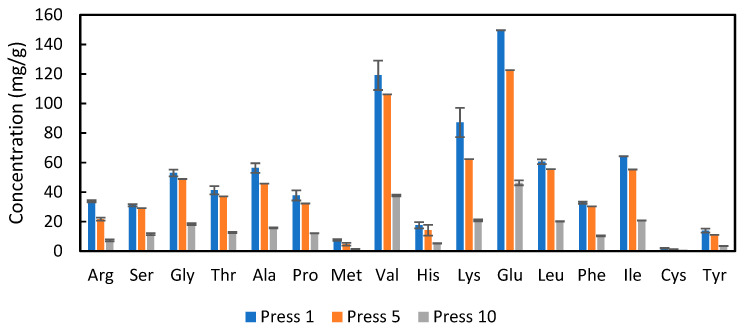
Concentration of amino acids on a dry weight basis for APC from press 1, 5 and 10, from the twin-screw extraction; error bars show standard deviations, n = 3.

To determine whether the alfalfa protein concentrate had a suitable amino acid profile and whether that profile was changed by repetitive pressing, analysis was conducted on presses 1, 5 and 10 from the twin-screw press. The results in Figure 6 show that the amino acids present in the highest concentrations were valine, lysine, and glutamic acid. As expected from the results in Figure 2, the concentrations of each amino acid declined as more presses were conducted. However, the overall profile (percentage of each amino acid) did not change markedly (see Table A2, Appendix B). 

**Figure 7:**
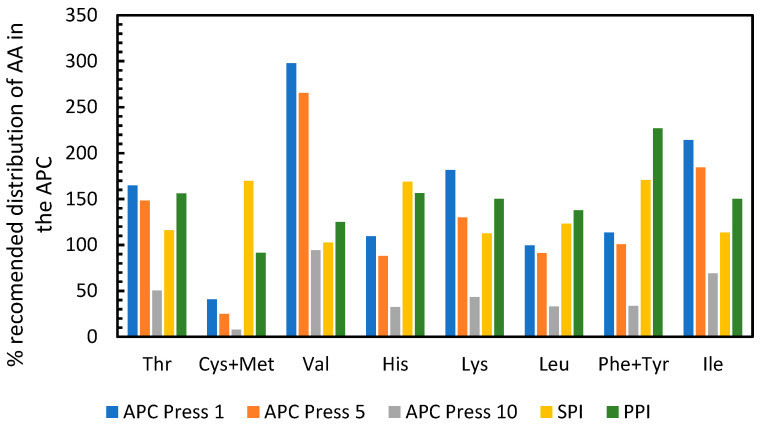
Percentage of each amino acid in APC and other protein sources normalized to the FAO recommended content of amino acids in protein sources. APC = Alfalfa Protein Concentrate, SPI = Soy Protein Isolate, PPI = Pea Protein Isolate. Data on PPI, SPI and reference values from Corgneau et al., 2019 [22].

The United Nations Food and Agricultural Organization (FAO) recommends that adult humans should consume 0.8 g of protein per kilogram of body weight per day [21]. Furthermore, the protein consumed should have a specific amino acid profile [21]. The APC produced here was compared to the FAO requirements and to two other popular plant based protein isolates, namely soy protein isolate (SPI) and pea protein isolate (PPI) in order to determine if APC had a similar quality. The data from SPI, PPI and the FAO reference values were obtained from Corgneau et al., 2019 [22]. For this comparison, the amount of each amino acid recommended by the FAO to be consumed per day was normalized to 100%. The data in Figure 7 shows that consuming 0.8 g of APC from press 1 per kg of body weight would supply markedly more of 7 out of the 10 essential amino acids when compared to the FAO recommendation. In particular, approximately three times the necessary amount of valine would be obtained. Consuming 0.8 g/kg bodyweight of the APC from press 5 would supply more than enough of six of the essential amino acids, whereas the APC from press 10 had a low amount of protein (Table A3, Appendix B), which results in insufficient supply of all amino acids. When the APC from press 1 is compared to soy protein isolate and pea protein isolate, it can be seen that APC is enriched in valine and isoleucine, has similar proportions of threonine and lysine, and lower proportions of cysteine + methionine, histidine and phenylalanine + tyrosine. The results suggest that the proteins in APC obtained from the first pressings would complement the amino acid profile of soy and pea protein isolates and give a profile that is acceptable for human consumption.

### 3.4. Color of the APC

**Figure 8:**
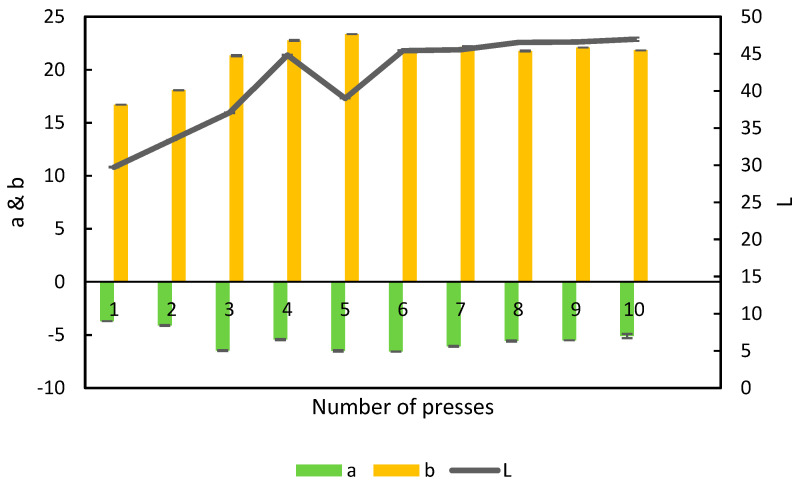
L-a-b color profile of alfalfa protein concentrate obtained in the 10 presses from the single-screw, n = 3, error bars show standard deviation.

L-a-b is the analysis of the color spectrum of a sample in three dimensions. “L” is light from 0 (dark) to 100 (white), “a” is from −100 (green) to 100 (red) and “b” is from −100 (blue) to 100 (yellow) [23]. When the color of the APC was measured, it was seen that the amount of green color increased from press 1–3 (Figure 8). This suggests an increase in extraction of the green chlorophyll to the APC. The leaf of the alfalfa has the highest concentration of protein and the highest concentration of chlorophyll in the plant [5,19]. This corresponds well with the amount of protein extracted presented in Table 2.

The amount of yellow color increased from press 1–5 and was thereafter more or less stable. Yellow pigment is speculated to be xanthophyll, which is found in most parts of plants, including alfalfa [7,24].

### 3.5. Properties of APC Produced with Single-Screw Press

The results in Figure 2 showed that protein was recovered in all 10 rounds of pressing. Furthermore, it was seen that the concentration of protein present (on a dry weight basis) was over 37% even in the APC produced from the tenth press (as seen in Table 2). However, the most soluble proportion of protein was found in the first three presses. In order to further determine the quality of the APC, the protein digestibility was analyzed. The protein digestibility was highest in press 1 at over 87% and declined to ~70% in press 5, after which it fluctuated in the range of ~60–65% (Table 2). This confirms that in the first pressings, the most soluble and easily digestible proteins are released, and that repeated pressings lead to release of poorly soluble, denatured or aggregated proteins as the alfalfa matrix is progressively broken down. It should be noted that the simple method used here most likely underestimates the digestibility, since it does not directly simulate the times and enzymes used in the mouth, stomach and gastric areas, as for example is the case with the Infogest assay. 

Consistent with the pattern of digestibility, is that the highest concentrations of the highly soluble simple sugars, glucose and fructose, were in the first presses, which then decrease with further pressing. Breakdown of the alfalfa matrix is also supported by the observation that the amount of insoluble fibers in the protein concentrate increased when repressing the pulp (Table 2). Interestingly the amount of soluble fibers was similar in all ten presses. 

The ash content was significantly higher in the first press (>8%) whereas it was around 3% in the following nine presses. The high ash content in the first press could be due to residual soil in the alfalfa, which was not washed away during the preparation of the raw material.

The total fat concentration was lowest in the first press and highest in the second. From the third to tenth press the total fat content was more or less similar. APC contains around 46% unsaturated fatty acids and 14% saturated fatty acids (i.e., values are as a percentage of the total fat). The main unsaturated fatty acid is alpha linoleic acid (34.1%) and the main saturated fatty acid is palmitic acid (12.3%) [6]. Since alpha linoleic acid is soluble in water and palmitic acid is not [25], it would be reasonable that many of the unsaturated acids are released in the second press after the plant material was partly opened up in the first press. A large reduction in the mass of pulp was observed from press 1, 2 and 3 (Table A1, Appendix A). From the fourth press until the 10th press, the decrease in the mass of pulp was low compared to first three presses. This might be a simple way to determine whether repetitive pressings should be stopped, or continued, when working with this in full scale. 

In both of the cases used here, more than 10 cycles of pressing are required in order to extract all protein from the plant material. However, much of the protein is insoluble. Considering the single-screw press, 2189 g of protein was present in 50.8 kg of fresh alfalfa. Given that 33% of this protein is expected to be soluble, this equates to a maximum yield of soluble protein of ca. 722 g. In 10 presses a total of 1041 g of protein was recovered (Table 2) and 314 g of this was soluble protein (derived from Table 2 and Figure 4). This suggests that almost 30% of the soluble protein was recovered, and more than half of this was recovered in the first two presses. 

## 4. Conclusions

Overall, the results indicate that small-scale laboratory trials with a twin-screw press can be scaled-up to a pilot scale single-screw press. However, the results also suggest that there is better maceration of the alfalfa with a twin-screw press. For scale-up it might therefore be beneficial to have a pre-maceration step, or to choose a screw design that macerates the plant material before pressing it, to maximize the protein yield. 

Using 10 pressing cycles, is undesirable due to the amount of water consumed, the time taken, the risk of oxidation and energy used. However, since alfalfa is harvested several times during a harvest season, it is concluded that two rounds of pressing could be used to recover up to 25% of the total protein and ca. 25% of the total soluble protein. This would result in an APC product with the highest digestibility and lowest fiber content. The resulting pulp could then be used for animal feed or other applications, such as mushroom production [26,27]. 

The protein concentrate produced here was observed to be dark green and to smell of grass. It is thus concluded that it contains many impurities and although it might be suitable as a supplement, it is not a product ready to be used in large amounts as an ingredient, which would be acceptable for human food production. Therefore, a full techno-economic evaluation of alfalfa protein concentrate production is premature at this point. More work is required to identify the best process for protein ingredient production from alfalfa. It is proposed that process development should proceed on two fronts: Optimizing protein recovery from alfalfa within two pressing steps and purification of the green protein.

## Figures and Tables

**Table 1:** Analyses of the raw alfalfa. Water content (%) n = 1, all others n = 3. Results are expressed in % wet weight.

Component	Protein (%)	Water (%)	Ash (%)	Fat (%)	Total (%)
Raw Alfalfa	4.3 ± 0.5	79.38	1.78 ± 0.21	0.25 ± 0.00	85.7

**Table 2:** Composition of the extracted APC in press 1–10 (all results expressed % DM). Values are averages and standard deviations, n = 3 except for insoluble fiber and soluble fiber, where n = 1.

Component\Press	1	2	3	4	5
APC obtained (g)	818.4	511	136.9	268.5	99.7
Protein (%)	43.12 ± 0.26	44.08 ± 0.83	47.40± 0.93	40.07± 0.68	42.14 ±1.06
Protein Digestibility (%)	87.36 ± 0.86	82.42 ± 1.30	76.20 ± 1.39	69.16 ± 0.49	70.43 ± 1.69
Insoluble fiber (%)	21.13	24.83	29.34	35.03	34.6
Soluble fiber (%)	1.67	1.21	2.17	2.22	2.41
Glucose (%)	3.15 ± 0.63	2.27 ± 0.48	1.79 ± 0.76	1.20 ± 0.65	2.14 ±0.00
Fructose (%)	2.29 ± 0.47	0.30 ± 0.30	0.04 ± 0.06	0.38 ± 0.53	0.00 ±0.00
Ash (%)	8.24 ± 0.28	3.15 ± 0.55	3.07 ± 0.37	2.72 ± 0.28	2.40 ±0.18
Fat (%)	1.25 ± 0.15	3.77 ± 0.86	2.46 ± 0.25	2.41 ± 0.12	2.76 ± 0.09
**Component\Press**	**6**	**7**	**8**	**9**	**10**
APC obtained (g)	126.8	134.8	133.6	120.3	120.8
Protein (%)	41.29 ± 0.77	39.08 ± 0.13	38.69 ± 0.59	38.24 ± 0.82	37.84 ±0.80
Protein Digestibility (%)	62.28 ± 1.39	61.84 ± 0.82	64.07 ± 0.35	63.72 ± 0.43	65.02 ± 1.16
Insoluble fiber (%)	39.36	41.56	42.16	44.94	45.6
Soluble fiber (%)	1.51	1.71	1.82	1.77	1.86
Glucose (%)	1.61 ± 0.12	1.00 ± 0.12	1.06 ± 0.07	0.77 ± 0.12	0.55 ±0.18
Fructose (%)	0.34± 0.36	0.38 ± 0.30	0.04 ± 0.06	0.17 ± 0.00	0.55 ±0.78
Ash (%)	3.16 ± 0.61	2.95 ± 0.50	2.95 ± 0.57	2.52 ± 0.25	2.70 ± 0.29
Fat (%)	2.56 ± 0.04	2.71 ± 0.16	2.31 ± 0.04	2.13 ± 0.16	2.25 ± 0.00

## Data Availability

The data presented in this study are available in the paper.

## Appendix A

**Table A1:** Masses (g) of the fractions produced during alfalfa processing with twin- and single-screw presses.

		Twin-Screw Press		
Press Number	Pulp	Green Juice	Brown Juice	Green Pellet
1	932	3589	3474	321
2	681	2059	1897	182
3	555	1415	1303	118
4	485	1138	1023	108
5	469	963	855	101
6	432	942	836	98
7	413	861	797	62
8	391	815	754	56
9	378	775	718	54
10	358	746	693	48
		**Single-Screw Press**		
**Press Number**	**Pulp**	**Green Juice**	**Brown Juice**	**Green Pellet**
1	16221	53131	47938	4834
2	12449	33703	42101	3959
3	9549	26566	25020	969
4	8769	34220	32297	1809.7
5	7494	17578	16814	693
6	7108	14514	13572	874
7	6587	15244	13696	1003
8	6183	13175	12091	1027
9	5849	12749	11789	974
10	5827	15900	14653	1113

## Appendix B

**Table A2:** Percentage of each amino acid in the APC produced in press 1, 5 and 10 from the twin-screw press.

Press	Arg	Ser	Gly	Thr	Ala	Pro
1	4.18	3.85	6.56	5.10	6.98	4.67
5	3.19	4.30	7.21	5.47	6.74	4.76
10	2.99	4.72	7.53	5.18	6.45	4.94
**Press**	**Met**	**Val**	**His**	**Lys**	**Glu**	**Leu**
1	0.92	14.76	2.17	10.79	18.54	7.51
5	0.68	15.67	2.08	9.19	18.09	8.19
10	0.59	15.46	2.13	8.54	18.99	8.23
**Press**	**Phe**	**Ile**	**Cys**	**Tyr**		
1	4.05	7.97	0.23	1.72		
5	4.47	8.16	0.16	1.63		
10	4.23	8.49	0.14	1.41		

**Table A3:** Total protein concentration in the APC obtained from the twin-screw press in presses 1–10, n = 3.

Press Number	1	2	3	4	5
**Protein (% DM)**	51.35 ± 0.08	49.16 ± 0.07	39.45 ± 0.41	36.75 ± 087	33.80 ± 0.09
**Press number**	**6**	**7**	**8**	**9**	**10**
**Protein (% DM)**	31.21 ± 0.29	28.07 ± 0.03	28.43 ± 0.09	26.69 ± 0.17	24.53 ± 0.12
